# Scaphoid Waist Internal Fixation for Fractures Trial (SWIFFT) protocol: a pragmatic multi-centre randomised controlled trial of cast treatment versus surgical fixation for the treatment of bi-cortical, minimally displaced fractures of the scaphoid waist in adults

**DOI:** 10.1186/s12891-016-1107-7

**Published:** 2016-06-04

**Authors:** Joseph Dias, Stephen Brealey, Surabhi Choudhary, Liz Cook, Matthew Costa, Caroline Fairhurst, Catherine Hewitt, Stephen Hodgson, Laura Jefferson, Kanagaratnam Jeyapalan, Ada Keding, Paul Leighton, Amar Rangan, Gerry Richardson, Claire Rothery, Nicholas Taub, John Thompson, David Torgerson

**Affiliations:** AToMS - Academic Team of Musculoskeletal Surgery, Undercroft (nr Ward 28), University Hospitals of Leicester NHS Trust, Leicester General Hospital, Gwendolen Road, Leicester, LE5 4PW UK; York Trials Unit, Department of Health Sciences, University of York, Heslington, York, YO10 5DD UK; Department of Radiology, University Hospital Birmingham, Mindelsohn Way, Edgbaston, Birmingham, B15 2WB UK; Nuffield Department of Orthopaedics, Rheumatology and Musculoskeletal Sciences, University of Oxford, The Kadoorie Centre, John Radcliffe Hospital, Oxford, OX3 9DU UK; York Trials Unit and NIHR RDS YH, Department of Health Sciences, Faculty of Science, University of York, ARRC Building, Heslington, York, YO10 5DD UK; Bolton NHS Foundation Trust, Trust HQ, Royal Bolton Hospital, 1st Floor, Minerva Road, Farnworth, Bolton, BL4 0JR UK; Department of Diagnostic Radiology, University Hospitals of Leicester, The Glenfield Hospital, Groby Road, Leicester, LE3 9QP UK; School of Medicine, University of Nottingham, Nottingham Health Science Partners, C-Floor South Block, Queens Medical Centre, Nottingham, NG7 2UH UK; South Tees Hospitals NHS Foundation Trust, The James Cook University Hospital, Marton Road, Middlesbrough, TS4 3BW UK; Centre for Health Economics, University of York, Heslington, York, YO10 5DD UK; Department of Health Sciences & NIHR Research Design Service for the East Midlands, University of Leicester, Centre for Medicine, University Road, Leicester, LE1 7RH UK; Department of Health Sciences, College of Medicine, Biological Sciences and Psychology, University of Leicester, Centre for Medicine, University Road, Leicester, LE1 7RH UK

**Keywords:** Scaphoid fracture, Screw fixation, Plaster cast, Union, Randomised controlled trial

## Abstract

**Background:**

A scaphoid fracture is the most common type of carpal fracture affecting young active people. The optimal management of this fracture is uncertain. When treated with a cast, 88 to 90 % of these fractures unite; however, for the remaining 10-12 % the non-union almost invariably leads to arthritis. The alternative is surgery to fix the scaphoid with a screw at the outset.

**Methods/Design:**

We will conduct a randomised controlled trial (RCT) of 438 adult patients with a “clear” and “bicortical” scaphoid waist fracture on plain radiographs to evaluate the clinical effectiveness and cost-effectiveness of plaster cast treatment (with fixation of those that fail to unite) versus early surgical fixation. The plaster cast treatment will be immobilisation in a below elbow cast for 6 to 10 weeks followed by mobilisation. If non-union is confirmed on plain radiographs and/or Computerised Tomogram at 6 to 12 weeks, then urgent surgical fixation will be performed. This is being compared with immediate surgical fixation with surgeons using their preferred technique and implant. These treatments will be undertaken in trauma units across the United Kingdom. The primary outcome and end-point will be the Patient Rated Wrist Evaluation (a patient self-reported assessment of wrist pain and function) at 52 weeks and also measured at 6, 12, 26 weeks and 5 years. Secondary outcomes include an assessment of radiological union of the fracture; quality of life; recovery of wrist range and strength; and complications. We will also qualitatively investigate patient experiences of their treatment.

**Discussion:**

Scaphoid fractures are an important public health problem as they predominantly affect young active individuals in the more productive working years of their lives. Non-union, if untreated, can lead to arthritis which can disable patients at a very young age. There is a rapidly increasing trend for immediate surgical fixation of these fractures but there is insufficient evidence from existing RCTs to support this. The SWIFFT Trial is a rigorously designed and adequately powered study which aims to contribute to the evidence-base to inform clinical decisions for the treatment of this common fracture in adults.

**Trial registration:**

The trial is registered with the International Standard Randomised Controlled Trial Register (ISRCTN67901257). Date registration assigned was 13/02/2013.

## Background

Scaphoid fracture is the most common carpal fracture and is an important public health problem as it predominantly affects young active individuals (mean age 29 years [1]) in their most productive working years. These fractures account for 2 to 7 % of all fractures [2]. However, 10 to 12 % do not unite when treated with plaster cast, with a higher incidence of non-union (14 to 50 %) in displaced fractures [3–5]. Non-union, if untreated almost inevitably leads to arthritis, usually within 5 years of injury [6, 7]. This can potentially disable patients at a relatively young age.

Recent systematic reviews [8–12] have found insufficient evidence from randomised controlled trials (RCTs) to inform clinical decisions on the treatment of scaphoid waist fractures. Eight small RCTs comparing surgery with non-operative management [12] could not establish whether patients who had surgical fixation of undisplaced or minimally displaced scaphoid fractures had better longer term outcomes. Surgery in these RCTs was shown to facilitate early return of function, but had a higher complication rate (between 9 and 22 %) compared with conservative management although the complications were usually minor [1, 13, 14]. The rate of union was similar between surgical and cast treatment with early fixation of those fractures that failed to unite [1]. A further study found similar outcomes at 10 years [15].

Despite insufficient evidence there is a rapidly increasing trend [16] for immediate surgical fixation compared to cast immobilisation for 6 weeks and fixing only those 10 to 12 % that fail to unite [1]. This current trend to fix fractures may be attributed to potential short-term benefits, but concerns remain about the lack of evidence on long-term benefits and additional risks from surgery, such as malunion, infection, implant related problems and avascular necrosis (AVN).

Hospital Episode Statistics (HES) for National Health Service (NHS) hospitals in England recorded a near doubling (1534, 1720 and 2582) of acute scaphoid fracture fixations for the years 2007/8, 2008/9 and 2009/10 respectively. In each of these three years we calculated an expected incidence of scaphoid waist fractures of 4140, 4169 and 4197 (population of 51.1, 51.4 and 51.8 million respectively in England in 2007, 2008 and 2009 and based on a rate of 81 acute scaphoid fractures/million population per year) [17]. We had to estimate this as currently the diagnosis of cases that do not receive surgical treatment, or those treated only in the outpatient setting, are not recorded in HES. The rate of surgical fixation [18] rose very slightly from 37 to 41 % from 2007/8 to 2008/9 but then increased sharply to 62 % in 2009/10. This trend of increasing intervention rate for these fractures emphasises the urgent need for this study.

Little is published on patient experiences after a scaphoid fracture and issues pertinent to recruiting participants into surgical RCTs [19, 20]. There is also poor information on the economic aspects [21] of this injury and its treatment.

These limitations, identified in the current evidence base, justify this adequately powered study. It will contribute to the evidence base for sound clinical decisions for the treatment of this common fracture in predominantly young adults.

## Methods/Design

### Design

This is a pragmatic, multi-centre RCT, investigating whether immediate surgical fixation of minimally displaced fractures of the scaphoid waist leads to better patient reported outcomes when compared to initial non-operative treatment with later surgical fixation of only those fractures that fail to unite. The study includes an economic analysis and has a nested qualitative study to explore patient experiences of their treatment. The objectives are listed in Table 1.

**Table 1:** SWIFFT trial objectives

Objectives	
1	Our primary objective is to determine the effectiveness of surgical fixation versus non-operative plaster cast treatment (with fixation of those that fail to unite, estimated as 10 % to 12 % of the total) of scaphoid waist fractures in adults. Outcome will be assessed using the PRWE (a patient self-reported assessment of wrist pain and function) at 52 weeks which will be the primary end point. The PRWE will also be completed at 6, 12, 26 weeks and 5 years. The power of the study permits identification of a clinically meaningful difference of 6 points in the PRWE.
2	To assess secondary outcomes of radiological union of the fracture at 52 weeks using radiographs and CT scans; recovery of wrist range and strength; return to work and recreational activities and; complications.
3	To conduct an economic analysis to investigate the cost-effectiveness of surgical fixation versus initial immobilisation in a plaster cast.
4	To qualitatively explore patient experiences of the fracture and its treatment; and investigate attitudes towards, and experiences of, participating in a surgical, clinical research trial.
5	To undertake a 5 year clinical review of all trial participants to determine the long-term consequences of cast immobilisation and internal fixation.

The trial is registered as ISRCTN67901257 and can be found by searching at http://www.isrctn.com/ at the time of registration we were not explicit that the pain and function sub-scales of the primary outcome would be included as secondary outcome measures, this had been included in the grant application.

### Setting

The Chief Investigator together with the British Society for Surgery of the Hand (BSSH) obtained agreements to participate from 17 hand units in NHS Hospital Trusts in the United Kingdom across a range of urban and rural areas. The pragmatic design of the trial and wide clinician involvement ensures immediate applicability and generalisability of the trial findings. Table 2 shows a list of all participating hospital sites that have been set up to recruit patients into the trial.

**Table 2:** SWIFFT participating hospital study sites

Study sites	
1.	Basingstoke and North Hampshire Hospital
2.	Royal United Hospital Bath NHS Trust
3.	Birmingham – Queen Elizabeth Hospital
4.	Bolton NHS Foundation Trust
5.	Brighton and Sussex University Hospitals NHS Trust
6.	Bristol Royal Infirmary
7.	Addenbrookes Hospital
8.	University Hospital Wales, Cardiff
9.	Royal Cornwall Hospital
10.	Coventry
11.	Gloucestershire Royal Hospital
12.	King’s College Hospital NHS Foundation Trust
13.	Leicester Royal Infirmary
14.	Royal Liverpool University Hospital
15.	Maidstone and Tunbridge Wells NHS Trust
16.	Medway Maritime Hospital
17.	Musgrove Park Hospital
18.	Newcastle upon Tyne Hospitals NHS Foundation Trust
19.	North Bristol NHS Trust
20.	Nottingham University Hospitals NHS Trust
21.	John Radcliffe Hospital, Oxford
22.	Peterborough City Hospital
23.	Derriford Hospital, Plymouth
24.	Poole Hospital
25.	Royal Preston Hospital
26.	Royal Berkshire Hospital
27.	Salford Royal Hospital NHS Foundation Trust
28.	University Southampton NHS Trust
29.	The Royal London Hospital
30.	The James Cook University Hospital, Teesside
31.	North Tyneside General Hospital
32.	Chelsea and Westminster Hospital NHS Foundation Trust
33.	Alexandra Hospital, Redditch
34.	Southport and Ormskirk Hospitals NHS Trust

### Study participants

Patients are eligible for this study if they

1. are skeletally mature and aged 16 years old or above
2. present at a participating site within two weeks of their injury
3. have a clear, unequivocal bicortical fracture of the scaphoid waist confirmed on a series of plain radiographs of the scaphoid which
  - does not involve the proximal pole (proximal fifth of the scaphoid) and
  - is minimally displaced with less than or equal to 2 mm step or gap on any view.

Patients will be excluded from this study if

1. their fracture has >2 mm displacement as these are likely to be unstable
2. they have a concurrent wrist fracture in the opposite limb
3. they have a trans-scaphoid perilunate dislocation
4. they have multiple injuries in the same limb
5. they lack mental capacity to comply with treatment or data collection
6. they are pregnant because radiation exposure would be contraindicated
7. they are not resident in the trauma catchment area of a participating site to allow follow-up.

### Trial interventions

#### Cast treatment

Control treatment is non-operative with immobilisation in a below elbow cast for 6 to 10 weeks, followed by mobilisation. Pragmatically we have not specified whether the thumb should be included in the cast as this does not affect union rate [3], but we will record the type of cast used. Early Computerised Tomogram (CT) will be obtained at the discretion of the treating surgeon if plain radiographs at 6 to 12 weeks raise the suspicion of non-union which we expect in 10 to 12 % of non-operatively treated patients. If non-union is confirmed on radiographs and/or CT scan, urgent surgical fixation will be performed. The surgical procedure and post-operative care will be similar to the surgical arm of this trial. This is the current standard non-operative pathway [1].

#### Surgery

Immediate surgical fixation avoids the need to immobilise the wrist in a cast and may accelerate return of function [22] but requires the individual to have surgery and be exposed to surgical risks.

Surgical treatment is by percutaneous or open surgical fixation with standard CE marked headless compression screws [14, 23, 24] which avoid the pressure effects of the screw head on articular cartilage. The surgical techniques are well described and are now standard [25–27]. We will not restrict the type of implant used but will record what screw was used. We will not specify the surgical approach or the postoperative care as most surgeons currently use some splintage for the first few weeks after surgery. It will be agreed at each recruiting site which surgeons will fix the scaphoid fractures and that these surgeons should use techniques with which they are familiar.

#### Rehabilitation

All patients randomised into the two groups will receive standardised, written physiotherapy advice detailing the exercises they need to perform for rehabilitation following their injury. All patients in both groups will be advised to move their shoulder, elbow and finger joints fully within the limits of their comfort. Those patients treated in a cast will perform range-of-movement exercises at the wrist as soon as their plaster cast is removed at the 6-week follow-up appointment if there are no concerns regarding bone union. Those patients who have the fracture fixed may begin wrist exercises as soon as comfort permits if they do not have a plaster cast or as soon as the cast is removed. In this pragmatic trial, any other rehabilitation input beyond the written information sheet (including a formal referral to physiotherapy) is at the discretion of the treating surgeon. However, a record of any additional rehabilitation input (type of input and number of additional appointments) is to be recorded at the 52 week follow-up.

### Outcome measures

Table 3 outlines the time points where various outcomes are assessed. These outcomes are described below.

**Table 3:**
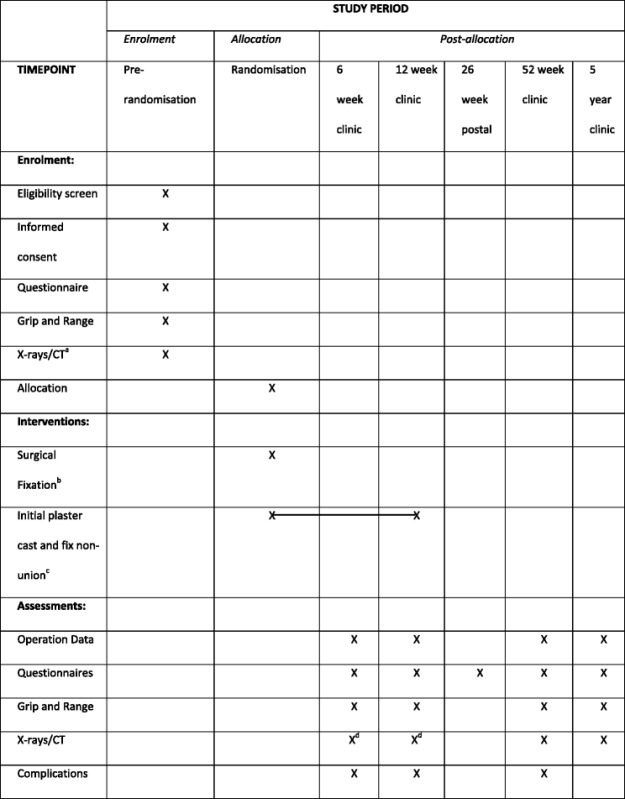
Timescale of enrolment, interventions and follow-ups for patients

^a^Where possible the CT should be done before randomisation, if this is not feasible it must be scheduled before surgery and must be done within two weeks of a patient’s injury

^b^Patients allocated to surgery must receive this within two weeks from when the patient presents to A&E or other point of contact (e.g. walk-in centre, cottage hospital)

^c^Patients are initially put into plaster cast for 6 to 10 weeks. If non-union is confirmed on X-rays/CT at 6 or 12 weeks urgent surgical fixation will be performed

^d^These X-rays/CT are those routinely collected whereas at the other time-points are compulsory

### Primary outcome

The primary outcome and end-point for the trial is the Patient Rated Wrist Evaluation (PRWE) total score at 52 weeks from randomisation.

### Patient rated wrist evaluation

The PRWE is completed at baseline for the time before and after injury, and at 6, 12, 26, 52 weeks and 5 years after randomisation. The PRWE is a 15-item questionnaire that is completed by the patient. It is a brief, reliable and valid instrument for assessing wrist pain and disability [28, 29]. Scoring for all the questions is on a 10-point, ordered scale ranging from ‘no pain’ or ‘no difficultly’ (0) to ‘worst ever pain’ or ‘unable to do’ (10). Two non-overlapping subscales are generated: pain and function and a total score can be computed on a scale of 0 to 100 (0 = no disability) where pain and function domains are weighted equally.

PRWE has been chosen as the primary outcome as patient reported functional outcomes are favoured for decision making and it allows assessment of both wrist pain and function.

### Timing of primary outcome

Two small RCTs [1, 14] of patients with fractures of the scaphoid have demonstrated that there is little change in objective and subjective outcomes between 26 and 52 weeks. For the 10 to 12 % of patients who are treated initially in cast but do not heal, surgery should be performed between 6 and 12 weeks from randomisation. Therefore, if assessed at 26 weeks this would leave only 14 to 20 weeks for healing and recovery to take place. To allow all patients the time to heal from surgery and recover from complications we have chosen 52 weeks as the primary end point.

### Secondary outcomes

#### Patient rated wrist evaluation

PRWE total scores at other time points (6, 12, 26 weeks and 5 years) as well as the PRWE subscale scores of pain and function will be secondary outcomes.

### Short form 12-item questionnaire (SF-12)

The SF-12 is a 12 item generic patient-reported outcome measure of physical and mental health, the population norms of which have a mean of 50 and standard deviation of 10; higher scores indicate better health [30]. The SF-12 is completed at 6, 12, 26 and 52 weeks and at 5 years to measure the potential broader consequences of a scaphoid fracture on both the participants’ physical and mental health.

### EuroQol (EQ-5D-3L)

The EQ-5D is a validated, generic patient-reported outcome measure covering five health domains (mobility, self-care, usual activities, pain/discomfort and anxiety/depression). We will use the original EQ-5D which contains three response options within each of the five domains [31, 32]. The use of this non-fracture-specific instrument will allow us to assess health-related quality of life outcomes in the health economic analysis. The EQ-5D has high validity and reliability in proximal humerus fractures [33] and hip fractures [34]. The EQ-5D is completed at baseline, 6, 12, 26 and 52 weeks.

### Bone union

The secondary outcome of bone union [35] is determined at 52 weeks (in line with the primary end point) using a CT scan and plain radiographs comprising posterior-anterior, lateral, semi 45° prone, semi 45°supine views and an elongated scaphoid view e.g. Ziter type view [36]. Union is defined as complete disappearance of the fracture line [3] on radiographs and complete bridging on CT scans [37–39] from those taken at baseline. We will identify partial union based on the proportion of the fracture plane traversed by bridging trabeculae on true sagittal and coronal formats of the scaphoid on CT. We are using CT to determine non-union as there is only poor to moderate inter-observer agreement (range of Kappa from 0.11 to 0.53) when determining the union of a scaphoid fracture on plain radiographs [40]. We will assess scaphoid fracture displacement on radiographs and on a CT scan [41] and determine malunion [42] on the 52 week CT scan (ratio of Scaphoid Height to Length ≥ 0.6) in the true sagittal axis of the scaphoid to assess any humpback deformity [43].

### Objective measures

We are measuring the range of movement [44] of both wrists using a goniometer and grip strength [45–48] of both hands using a calibrated Jamar dynamometer at baseline, 6, 12, 52 weeks and 5 years.

The measurements will be done with the subject seated, arm by the side, elbow bent at 90° and the wrist in neutral position for rotation [49]. The second setting on the Jamar dynamometer will usually be used but patients with large hands may need to use the third setting. This reflects common and evidence-based practice in assessing grip strength [50]. The Beighton Joint Laxity Score (excluding the thumb count for the injured wrist) will be recorded at baseline to measure hypermobility of joints [51]. These assessments will be standardised across participating sites using an instruction manual.

### Return to work and recreational activities

This will be established through patient self-report on the number of days off work and ability to perform usual activities when at work and when performing unpaid recreational activities. This will be recorded at 6, 12, 26 and 52 week follow-up.

### Complications

Expected and unexpected complications will be recorded at the 6, 12 and 52 week visit. The expected complications include:

1. Infection, defined as for the “Surgical Site Infection” audit [52].
2. Delayed wound healing, defined as any wound that has not healed by two weeks.
3. Complex Regional Pain Syndrome (CRPS), defined as puffy painful swelling of the whole hand restricting full tuck of the fingers at 2 weeks.
4. Nerve events (hypoaesthesia or numbness in the territory of the palmar cutaneous branch of the median nerve, superficial division of the radial nerve or the median nerve).
5. Vessel events (large (>2 cm) haematoma in the line of the radial artery).
6. Screw related complications (protrusion of either end into the adjacent joint, fracture or bending of the screw, a radiolucent halo around any part of the screw > 1 mm, screw backing out or moving).
7. Degenerative change in the adjacent joints [53].
8. Avascular necrosis (AVN) of the proximal pole of the scaphoid.

### Five year clinic review

The long-term consequences of cast immobilisation and internal fixation have not been adequately determined in RCTs. Therefore, at five years after their original injury, all remaining trial participants will be asked to attend a follow-up visit at a participating hospital for a clinical and radiographic follow-up. The clinical examination will include inspection and evaluation of scar sensibility when applicable, palpation for tenderness, measurement of joint movement with a goniometer, as well as measurement of grip strength and pinch strength [15].

Participants will complete a questionnaire that asks about perceived hand problems (e.g. weakness of wrist, reduced range of movement) as well as the primary outcome measure, the PRWE, and EQ-5D.

### Participant timeline

**Fig. 1:**
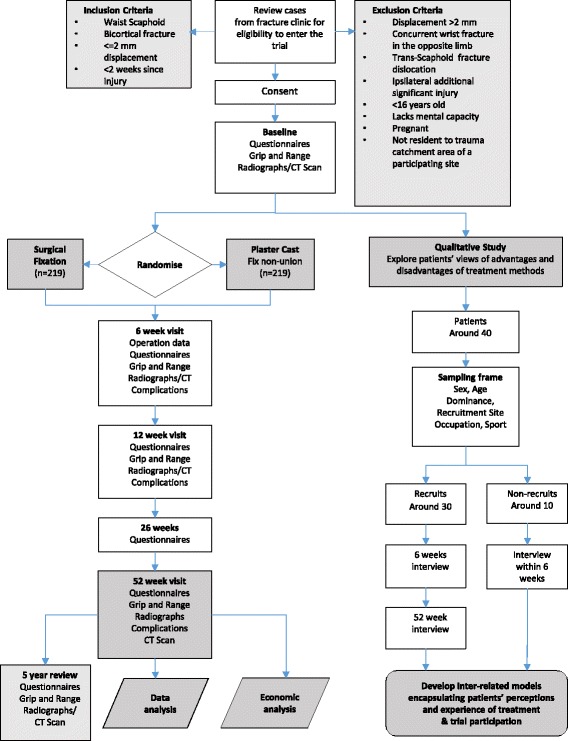
Flow of participants in the SWIFFT trial

Figure 1 illustrates the process of enrolling participants into the study, the interventions being compared, and timing of assessments and hospital visits for the participants in the trial.

### Sample size

For surgery to justify its increased costs and the exposure to risk, it must result in greater or quicker improvement in patients’ wrist symptoms and function compared with non-operative management. We judge that a 6 point improvement in the PRWE in the surgery group (compared to the controls) would be a minimal clinical important difference. We estimate the standard deviation of PRWE at 52 weeks to be 20 points from the PRWE User manual [54]. This figure is reported for distal radius fracture rather than scaphoid fracture at 6 months. The only published evidence for scaphoid fracture implies a standard deviation in the range of 8 to 10 points [15]; however, this estimate was at a median of ten years after the patient’s injury. To be conservative we have chosen the estimate of standard deviation to be 20, which gives a standard effect size of 0.3 for the 6 point PRWE difference.

We will use a superiority design to observe an effect size of 0.3 at 80 % power using a 2-sided 5 % significance level requiring 350 participants in total. After allowing for 20 % attrition we need to recruit and randomise 438 participants (219 surgery and 219 controls). The estimate of attrition is likely to be realistic given that four RCTs (three studies were single centre and one study had two centres) included in a systematic review of the treatment of scaphoid fractures found response rates for completion of patient-reported functional outcomes between 77 and 100 % [12].

To minimise attrition we will exclude the rare patient in this population who lacks mental capacity and therefore unlikely to comply with treatment or data collection. Active and systematic follow-up of all randomised participants will be conducted at 6, 12, 26 and 52 weeks when we will arrange for the questionnaire to be completed when the participant attends the clinic. An additional 5 year review is also planned. We will monitor the completion of questionnaires at clinics and share retention figures with each trial site blinded by centre.

At 52 weeks a £40 payment will be made to patients who attend the clinic. Concurrently we will employ a proven postal strategy for the return of questionnaires. This will include the use of reminder letters after 2 and 4 weeks and the option for completion of an abridged questionnaire (a minimum of the primary outcome and EQ-5D) via telephone after 6 weeks. At 52 weeks only (the primary time-point for the study), in addition to the 6 week telephone call we will write to non-responding patients to ask them to complete the PRWE to help maximise return of the primary outcome. We will still call the patient to complete the remainder of the questionnaire over the telephone. At 26 weeks, when the patient does not need to attend for a hospital appointment, we will include an unconditional incentive payment of £5 with the postal questionnaire. We will also circulate a regular newsletter and update the trial website (http://www.swifft.co.uk/), to keep the participants informed of study progress and engaged with the trial. A trial ‘tagline’ will be placed on postal envelopes to patients to highlight the importance of patients’ involvement in the research. At the five year follow-up, participants will receive £80 to attend hospital for their clinic review which should cover time off work, travel and parking costs. Finally, to minimise attrition bias, as there is randomised evidence in a recent systematic review that the return of postal questionnaires can be improved when patients are included in a prize draw [55], those patients who return the questionnaire at 26 weeks could win an iPad worth £500. When patients attend their hospital clinic appointment at 52 weeks and 5 years, they will be entered into additional prize draws to win an iPad worth £500.

### Recruitment

Strategies for achieving adequate participant enrolment to reach target sample size include seeking advice from our patient focus group, sharing best practice with our Research Nurses, and bi-annual discussion with our Principal Investigators at the scientific meetings of the BSSH. Table 4 shows the project plan and milestones.

**Table 4:** Time schedule of the study project plan

Time period (month)	Activity
1–3	Complete local R&D approval and set up for 4 sites
4–6	Initiate early recruitment (internal pilot study at 4 sites and continue R&D approval for other sites)
7–36	Main recruitment for trial
37–48	Complete final 12 month follow-up
49–54	Analysis and write up of main HTA monograph
55–63	Preparation for 5 year clinic review
64–96	Conduct 5 year clinic review
64–69	Internal pilot follow-up for 5 year review
70–72	Three months to follow-up the last participant to be followed-up for the internal pilot
73–75	If internal pilot is unsuccessful, to complete analysis and write up the report
97–105	Allow 9 months to complete clinics and prepare analysis
106–108	Complete analysis and write HTA addendum monograph

Hospital staff will be provided with training at the Site Initiation Visits and a Trial Site Manual to ensure adherence to the delivery of the interventions in the trial. During the trial, training and reminders will be implemented using e-mail bulletins, face-to-face meetings with the PI’s at BSSH conferences and a training day with Research Nurses. In addition the Trial Co-ordinators will provide support and guidance to staff when required (e.g. when new staff join or replace existing site staff) and will seek clinical guidance from the CI when necessary.

To assess adherence with the trial protocol, sites will be asked at the 6 and 12 week hospital visits to complete a ‘Treatment Confirmation Form’. This will be used to record the treatment that the patient received after randomisation and reasons for any change in treatment. It also asks whether non-union is suspected for patients randomised to conservative treatment. This will allow the trial team to check with the hospital site that surgical fixation is being offered to these patients. Trial participants are also asked at their 12 week follow-up how many of the home exercises they have performed and how useful they found the leaflet detailing exercises they need to perform for rehabilitation.

### Randomisation

The Research Nurse will identify potentially eligible patients presenting in Accident and Emergency (A&E) Departments, or referred from various sources (e.g. walk-in centre, cottage hospital), and at fracture clinics. The orthopaedic surgeon will confirm eligibility and invite the patient to consider joining the study. The Research Nurse or clinician will then provide an information sheet and answer any questions. The patient will be asked whether they agree to consent at that time or are offered up to 48 h to discuss the study and their participation with family or friends before deciding.

When patients have given consent their baseline forms will be completed and randomisation done. The Research Nurse or recruiting clinician will contact York Trials Unit, either by telephone or via the internet, to access the secure randomisation service. This will ensure treatment concealment and unbiased allocation. Once eligibility is confirmed, patients will be randomised (via a secure, computer generated allocation sequence) to receive immediate surgical fixation or immobilisation of the wrist in a cast.

Patients and their clinician will be informed of the allocations. As the trial is pragmatic and compares surgery with initial cast treatment, blinding of participants and clinicians to treatment allocation is not possible. When possible, the surgeon will take no part in the postoperative subjective assessment of patients. The statistical analysis will also be performed blind as far as possible. We will mitigate against bias by ensuring that all radiographs and CT scans are assessed by two independent consultant musculoskeletal radiologists and a consultant orthopaedic surgeon.

**Fig. 2:**
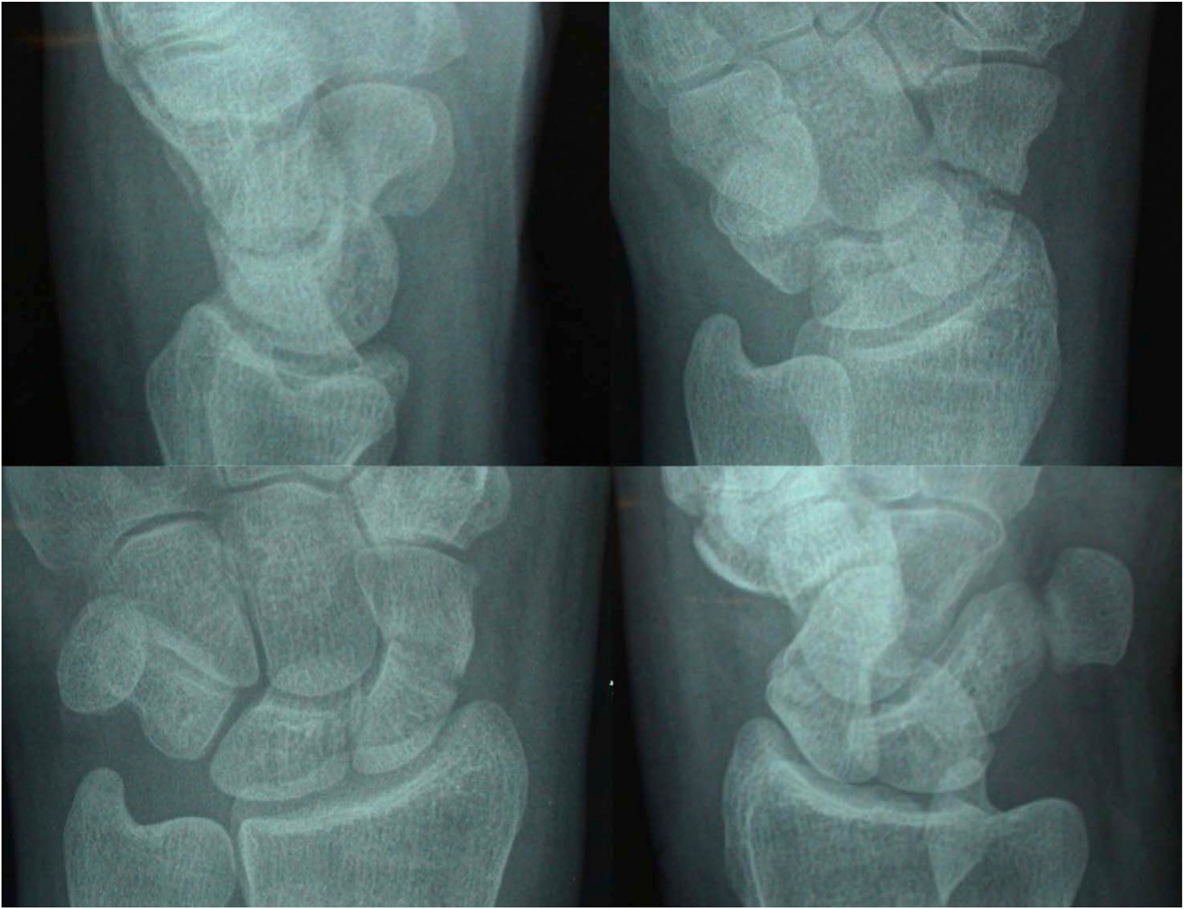
Radiographic views of a scaphoid fracture. Four radiographic views shown here, and a fifth elongated scaphoid view, establish the presence of a “clear” and ”bicortical” fracture of the waist of the scaphoid. Such patients are eligible for the SWIFFT trial. These radiographs also help determine whether the fracture is displaced for blocked randomisation after obtaining consent

As the non-union rate for displaced fractures is 14 % compared with 10 % for transverse undisplaced fractures [3, 5, 56], randomisation will be stratified by the presence or not of displacement of a scaphoid fracture as seen on radiographs [5, 56] Within strata, a sequence of confidential random block sizes will be used to generate the block allocation sequence. To avoid predictability of the randomisation we will not stratify by site. The radiographic views to assess displacement should include the semi 45° prone, semi 45°supine and an elongated scaphoid view e.g. Ziter [36] type view (Fig. 2). Displacement is defined as a step greater than 1 mm and less than or equal to 2 mm or a gap greater than 1 mm and less than or equal to 2 mm as seen on the radiographic views. This magnitude of displacement will avoid compromising surgeon equipoise for non-operative treatment as displacement >2 mm suggests significant instability and therefore the need for surgical intervention.

### Data management

Case Report Forms will be used to record all the information required from the protocol. Essential Trial documentation which individually and collectively permits evaluation of the conduct of a clinical trial and the quality of the data produced will be kept within the Trial Master File and Investigator Site Files. The Sponsor will ensure that this documentation is retained for a minimum of five years after the conclusion of the trial and a minimum of 20 years in electronic format in accordance with guidelines on Good Research Practice. Paper data will then be disposed of securely and electronic data will be anonymous of identifiable information.

All study-related information will be stored securely in the co-ordinating centre at the University of York or at an alternative secure off-site facility. All electronic records will be stored on a password-protected server. All participant data will be identified by a coded ID (identification) number to maintain participant confidentiality. All participant information will be stored in locked cabinets in areas with restricted access. Data on trial participants’ X-rays and CT scans will be stored securely at The University Hospitals of Leicester NHS Trust. Participants’ data may be reviewed by authorised persons on the research team or other authorised people to verify that the study is being carried out correctly all of whom will have a duty of confidentiality. Trial participants will give permission for this authorised review of their data at the time of consent. All names and other identifying information will be removed before the data is analysed and the results presented to the medical community at conferences and in scientific journals.

The Data Monitoring and Ethics Committee (DMEC) will be the only body to have access to the unblinded comparative data from the trial. The role of its members is to monitor these data and make any recommendations to the Trial Steering Committee (TSC) on whether there are any ethical or safety reasons why the trial should not continue. The TSC will provide overall supervision for the trial on behalf of the Sponsor and Funder.

### Statistical analysis plan

A detailed analysis plan will be agreed with the Data Monitoring and Ethics Committee at an early stage of the study, before all of the data has been collected. Any subsequent amendments will be clearly stated and justified. All analyses will be conducted on an intention to treat basis, including all available randomised participants in the groups to which they were allocated. Analyses will be conducted using 2-sided significance tests at the 5 % significance level (unless otherwise stated). The statistician conducting the analyses will remain blind to treatment group until all data summaries and results are finalised. For some variables the type of data reveals treatment allocation (e.g. complication data), hence relevant analyses will be conducted by a second statistician.

The flow of participants through each stage of the trial will be presented in a CONSORT flow diagram [57]. PRWE scores will be summarised descriptively (number, mean, standard deviation, median, interquartile range, minimum and maximum) at each time point by treatment group and overall. PRWE at baseline will be collected for the time before and after injury. Mean pre-injury PRWE scores will be presented in total and for each treatment group and compared descriptively to PRWE scores post-injury at baseline and all other follow-up time points.

Our primary analysis will compare total PRWE scores between treatment groups at 52 weeks using a covariance pattern mixed model incorporating all post-randomisation time points, where effects of interest and baseline covariates are specified as fixed effects, and the correlation of observations within patients over time (random effect) is modelled by a covariance structure. The outcome modelled will be PRWE at 6, 12, 26 and 52 weeks, predicted by treatment group, time, treatment group-by-time interaction and adjusting for age, fracture displacement (undisplaced vs. minimally displaced) and hand dominance. Estimates of the difference between treatment groups in total PRWE scores will be derived at all time points with 95 % confidence intervals and *p*-values. The primary end point will be the treatment effect estimate at 52 weeks. This model will naturally include all patients who provide data for the baseline covariates, and valid PRWE data for at least one post-randomisation follow-up time point.

The impact of missing PRWE outcome data will be minimised to some extent by using the mixed model, which allows the inclusion of intermittent responders in the primary analysis. PRWE scores for complete and intermittent responders will be compared descriptively. The impact of missing data will additionally be assessed using multiple imputation by chained equations. Missing outcome and covariate data will be predicted by age, fracture displacement, hand dominance, available PRWE data at other follow-up time points, and any baseline covariates found to be predictive of missing 12 month outcome data.

All secondary outcomes will be summarised descriptively. The following outcomes will be analysed using the same methods as the primary analysis adjusting for the same covariates: pain and disability subscales of the PRWE, physical health and mental health component summaries of the SF-12 and range and grip strength. Where available, baseline values of the dependent variable will also be included as a covariate in the models. The presence of any complication assessed by clinical examination up to 52 weeks will be analysed by logistic regression (sufficient numbers permitting). Complications will be defined as medical, surgical or plaster cast related. Union will be assessed as a percentage (0–100 %) and categorised as: total non-union [0 %]; slight union [>0–20]; partial union [>20–70]; mostly united [>70–<100]; and complete union [100 %]. Summary statistics for union will be presented at each time point by trial arm. Union will be dichotomised into a ‘Probably need surgery’ group [0–20 %] and a ‘Probably do not need surgery’ group [>20–100 %] and analysed using a logistic regression adjusting for treatment group.

Two subgroup analyses will be undertaken: one exploring patient preferences (surgery, plaster cast, no preference) and the second exploring the type of fracture displacement (undisplaced, displaced), for any differential effect of the trial treatments in these subgroups. Each baseline factor (preference or displacement) and its interaction with the randomised treatment group will be added to the primary analysis model. Our expectation is for patients who preferred surgery to benefit more from surgery over plaster cast treatment and conversely for patients who preferred plaster cast to benefit more from plaster cast than surgery. For patients with a displaced fracture we expect them to benefit more following surgery than patients whose fracture is undisplaced. Since the trial is not powered for these subgroup analyses, any inferences will be made with caution.

The number of adverse events experienced by each participant and the total number of events overall will be summarised for each treatment group.

#### Interim analysis

There are no planned interim analyses for the trial or stopping guidelines. There will, however, be an internal pilot study from which the data will contribute to the final analyses. The primary reason for this pilot study will be to check the assumptions about recruitment and feasibility of the trial. The DMEC and TSC will review the pilot data and recommend whether any changes are required to the trial team and whether the trial should continue or not. Furthermore, as we did not have data on the standard deviation of our primary outcome at one year, we will estimate this for the patients recruited into the internal pilot study. This will inform the potential need to increase the planned sample size as recommended by the independent DMEC.

### Cost-effectiveness analysis

The economic evaluation will assess the relative cost-effectiveness of surgical fixation compared with plaster cast treatment. Costs and health outcomes associated with the interventions will be collected during the 1-year trial period. However, the trial data is unlikely to provide all the evidence to inform the decision on whether surgical treatment represents a cost-effective option to the NHS. Therefore, these costs and outcomes will be extrapolated and modelled over a longer time horizon than captured by the trial (e.g. lifetime of the patient) if this is appropriate given the results of the trial. The additional data from the 5-year follow-up review on the long-term consequences of cast immobilisation and internal fixation, which are not adequately captured in RCTs, will be used to update the model results once available.

Detailed information will be collected on the costs of surgical fixation, including time in theatre, drugs and hospital bed usage, and the costs associated with plaster cast treatment. The impact of the two treatments on subsequent morbidity costs will be assessed. The use of hospital readmissions, outpatient attendances, general practice, community and personal health services will be collected during the various follow-up points through administered questionnaires. The primary perspective of the analysis will be that of the NHS and Personal Social Services, consistent with that used by the National Institute for Health and Care Excellence [58]. Private expenditures related to treatment will also be recorded and these costs will be included in a secondary analysis.

Health outcomes will be expressed in terms of the quality-adjusted life year (QALY) using the EQ-5D data collected at baseline, 6, 12, 26, and 52 weeks follow-up. The EQ-5D scores will be converted into QALYs using area under the curve analysis [59].

Cost and QALY data will be synthesised to generate an incremental cost effectiveness ratio (ICER) [60]. Multivariable regression analyses will be used to assess heterogeneity in costs, QALYs and cost effectiveness.

The 5-year follow-up review will facilitate an additional analysis that examines the relationship between outcomes reported at 1 year and 5 years. If considered appropriate, structural equation modelling [61] will be used to determine the factors that predict outcomes at 5 years and to assess the predictive performance of outcomes at 1-year. This may also be used to assess the cost-effectiveness of following patients up at 5 years post-surgery in general practice.

### Qualitative study of patient experience

The qualitative data collected in the nested study will be used to generate a model (or models) which reflect patient experiences of wrist fracture and treatment, and which identify difficulties and advantages of the different treatment options. Such models are likely to focus upon personal and lifestyle attributes as well as physical recovery, and to incorporate a range of non-clinical factors which are not routinely considered in clinical interactions. Insight into participation in a clinical trial will also be generated.

Sampling to the nested qualitative study will be purposive to include men and women from different trial sites, of different ages, occupations and leisure/sporting activities, and those with scaphoid fractures on their dominant and non-dominant sides. The sample will be drawn primarily from those individuals recruited to the trial (n to be determined by concerns for data saturation, see below). In addition, up to ten individuals who decline participation in the trial will be interviewed to explore their experience of fracture and their reasons for not taking part in the RCT. All participants will be interviewed within 6 weeks of their treatment and those in the trial will be interviewed again following collection of clinical and other data at the primary end-point (52 weeks).

All interviews will be semi-structured with open questions used to guide a discussion of a patient’s experience of treatment, their opinions about treatment benefits and drawbacks, their reflections upon wrist fracture and recovery, and their attitudes towards participating in clinical research. All interviews will be digitally recorded and transcribed in full.

Following the conventions of the constant comparative method, [62, 63] data analysis will be carried out alongside data collection, with interviews transcribed and analysed in batches before further data are collected. In this way, the process is iterative with models and theories developed from ‘within’ the interviews rather than from existing theory or clinical practice and tested or refined in the collection of more data.

These models will be further tested and *constantly* refined as new data are considered. Data collection, and analysis, ceases when no new themes or ideas are present in the interview data, and when the model of patient experience is stable and no longer growing or evolving. This point is known as data saturation [64], previous research suggests that this is often reached with as few as 10–13 interviews [65] – we are conducting around 40 interviews to include at least 15 from each treatment arm to enable data saturation. Data from those individuals not in the main trial will be considered alongside this data, and considered separately to inform practical concerns of trial recruitment.

Within this study we would expect to generate up to 4 interrelated models of patient experience: i) reporting patients’ experiences of wrist fracture, its impact upon their lifestyle, everyday functioning and their recovery; ii) reporting the benefits and difficulties associated with surgical fixation; iii) reporting the benefits and difficulties associated with plaster cast treatment and, iv) reporting experiences and attitudes towards involvement in surgical, clinical research.

### Adverse event management

Adverse events (AEs) related to the scaphoid fracture injury and its treatment during the 12 months after randomisation will be recorded by site Investigators and the categorisation of causality and expectedness confirmed by the Chief Investigator. AEs that may be expected with this injury to the wrist or a consequence of the trial treatments that do not need to be reported to the Research Ethics Committee (REC) include infection, delayed wound healing, CRPS, nerve or vessel events, screw related complications, fracture of scaphoid tuberosity, and chondrolysis. There are also adverse events specific to the plaster cast which are expected and do not need reporting to REC: soft cast/broken cast that leads to movement of wrist, pressure sores, CRPS, nerve compression, or pain due to tight cast. Movement in a cast is an untoward event as it can mean the fracture is not properly immobilised which can result in failure of the fracture to unite.

Serious adverse events that are confirmed to be *related* to the research and are *unexpected* will be reported to REC. All AEs will be routinely reported to the TMG, DMEC, and Sponsor. The DMEC will be responsible for reviewing related *and* unexpected serious adverse events.

All AEs that are unresolved at initial reporting will be reviewed by the Chief Investigator a month later to ensure that adequate action has been taken and progress made to manage the adverse event. Additional reviews at one month intervals will be conducted when necessary until the Chief Investigator decides that no further reporting is required.

The Chief Investigator will also be informed, by the reviewers of the X-rays/CT scans collected for the study, of any abnormalities identified. The Chief Investigator will judge whether the abnormality is clinically important and could impact on patient safety (e.g. a protruding screw). The need to notify the Principal Investigator of the site, and whether to record this as an AE, will also be considered. No actions or treatments will be discussed between the Investigators.

### Quality control

The University Hospitals of Leicester NHS Trust will be the Sponsor for this project. This study will be fully compliant with the Research Governance Framework and Medical Research Council Good Clinical Practice Guidance. If a patient wishes to complain formally, they will be advised to do this through the usual NHS Complaints Procedure. If a patient is harmed and this is due to someone’s negligence then they may have grounds for legal action or compensation against the Sponsor (in respect of harm arising out of participation in the trial) or the NHS (in respect of any harm which has resulted from the treatment received).

Review of core trial processes will be undertaken by the Trial Management Group (TMG) on a quarterly basis which includes representation from the Sponsor. These meetings focus on aspects of patient recruitment (e.g. enrolment, consent, eligibility); allocation to study groups; adherence of the trial interventions to the protocol; monitoring of adverse events and reasons for patient withdrawal; and retention of trial participants. When necessary the review will be undertaken at a recruiting site level and information feedback to the Principal Investigator and Research Nurses at each site. Independent review of the trial processes is undertaken every six months by the DMEC and TSC. These committees assist the TMG with their audit of trial processes and advise on strategies to preserve the integrity of the trial.

### Protocol modifications

Important protocol modifications are those that are likely to affect to a significant degree: the safety, physical or mental integrity of the subjects of the study; the scientific value of the study; or the conduct or management of the study. These substantial amendments will be submitted to REC for approval having been agreed with: the Funding Body, Sponsor, TSC, DMEC, TMG and the Research Governance Committee for the Department of Health Sciences, University of York. Minor modifications to the protocol will be agreed with the TMG and Sponsor before submission for approval to REC. All amendments will be implemented in the NHS organisations in agreement with the guidance of the Health Research Authority. Trial participants will be written to, if necessary, to explain any changes. All amendments whether substantial or not will be listed in the published Final Report to the Funding Body.

### Dissemination policy

This protocol is being made publically available. It is planned for the full Trial Report up to the 52 week follow-up to be submitted to the Funding Body and for publication in a peer-reviewed journal.. The full trial report will be open access and made available as a permanent archive in the NIHR Journals Library. At the time of publishing the protocol there was no plan to make the anonymised participant level dataset and statistical code for generating the results publicly available. After publication, however, of the main trial findings, an external request that is made for this data and code will be agreed by the TMG and confirmed with the Sponsor and Funding Body.

The criteria for authorship and contributorship will be taken from the International Committee of Medical Journal Editors [66]. Those who did not design the study or contribute to drafting the work but were involved in the trial conduct (e.g. staff at recruiting sites) will be acknowledged as collaborators. When a journal permits we will list all authors rather than use a group name. There will be a designated writing group for each publication and one or more lead writers who convene the group. Any member of the trial team can propose a publication to the Chief Investigator, Trial Manager and Senior member of York Trials Unit. All members of the trial team will be informed of the proposal who will suggest whether they consider themselves to be a potential author, contributor or neither. The Chief Investigator, Trial Manager and Senior member of York Trials Unit will then agree on this. Order of authorship will be determined by individuals’ completion of the Author Order Form which weights their contribution to elements of preparing the manuscript. Any individual who feels the order of the authorship does not reflect their input will notify this to the Chief Investigator, Trial Manager and Senior member of York Trials Unit. There are no plans to use professional medical writers to assist with the preparation of trial reports or publications.

## Discussion

Scaphoid fractures are an important public health problem as they predominantly affect young active individuals in the more productive working years of their lives. Non-union, if untreated, can lead to arthritis which can disable patients at a very young age. There is a rapidly increasing trend for immediate surgical fixation of these fractures but there is insufficient evidence from existing RCTs to support this. The SWIFFT Trial is a rigorously designed and adequately powered study which aims to contribute to the evidence-base for informing clinical decisions for the treatment of this common fracture in adults.

## Abbreviations

AVN, avascular necrosis; BSSH, British Society for Surgery of the Hand; CONSORT, Consolidated Standards of Reporting Trials; CRPS, complex regional pain syndrome; CT, computerised tomogram; DMEC, Data Monitoring and Ethics Committee; EQ-5D, EuroQol 5 Dimensions; HES, Hospital Episode Statistics; ICER, incremental cost effectiveness ratio; ISRCTN, International Standard Randomised Controlled Trial Number; NHS, National Health Service; PRWE, Patient Rated Wrist Evaluation; QALY, quality-adjusted life year; RCT, Randomised Controlled Trial; REC, Research Ethics Committee; SF-12, Short Form 12-Item Questionnaire; SWIFFT, Scaphoid Waist Internal Fixation for Fractures Trial; TMG, Trial Management Group; TSC, Trial Steering Committee
